# Prediction of Atrial Fibrillation Risk Through the Integration of Genetic Information and Artificial Intelligence‐Based Electrocardiogram Data

**DOI:** 10.1002/joa3.70463

**Published:** 2026-09-24

**Authors:** Pil‐Sung Yang, Hanjin Park, Oh‐Seok Kwon, Je‐Wook Park, Daehoon Kim, Hee Tae Yu, Tae‐Hoon Kim, Jae‐Sun Uhm, Boyoung Joung, Hui‐Nam Pak

**Affiliations:** ^1^ Yonsei University College of Medicine Yonsei University Health System Seoul Republic of Korea

**Keywords:** artificial intelligence–electrocardiogram (AI‐ECG), atrial fibrillation, CHARGE‐AF, multimodal integration, polygenic risk score (PRS), risk prediction

## Abstract

**Background:**

Accurate prediction of atrial fibrillation (AF) is essential for prevention. The CHARGE‐AF score, based on routinely available clinical factors, provides a practical tool for estimating AF risk but has limited predictive accuracy. This study aimed to improve AF risk prediction by integrating artificial intelligence (AI)–based electrocardiogram (ECG) analysis of age and sex with genetic information in addition to established clinical risk factors.

**Methods:**

We analyzed 39 478 UK Biobank participants without prior AF. A polygenic risk score for AF (AF‐PRS), the AI‐ECG age gap (AI‐ECG–predicted age minus chronological age), and AI‐ECG–predicted sex mismatch were evaluated on top of the CHARGE‐AF model. Model performance was compared across sequential prediction models.

**Results:**

Over a median follow‐up of 2.7 years (interquartile range, 1.7–4.2; maximum, 6.7), 533 participants (1.3%) developed AF. AF‐PRS (HR 1.61, 95% CI: 1.48–1.76) and the AI‐ECG age gap (HR 1.37, 95% CI: 1.24–1.51) were independently associated with incident AF. Compared with CHARGE‐AF alone (C‐index 0.708, 95% CI: 0.686–0.730), adding AF‐PRS improved discrimination (C‐index 0.743; Δ0.035, 95% CI: 0.021–0.049; *p* < 0.001). Adding traditional ECG parameters did not enhance performance, whereas incorporating AI‐ECG features (age gap and sex mismatch) into the combined clinical and genetic model provided additional gain in discrimination (C‐index 0.754; Δ0.045, 95% CI: 0.028–0.062), reclassification (net reclassification improvement 0.408), and discrimination improvement (0.007; all *p* < 0.001).

**Conclusions:**

Integrating genetic risk and AI‐ECG–derived features enhances AF prediction beyond an established clinical model, supporting the use of combined digital and genetic biomarkers for risk stratification.

## Graphical abstract

In 39 478 UK Biobank participants, integrating atrial fibrillation (AF) polygenic risk and AI‐ECG–derived features with the CHARGE‐AF clinical risk model significantly improved prediction of incident AF. These findings support the complementary value of genetic and AI‐ECG information for more refined AF risk stratification.

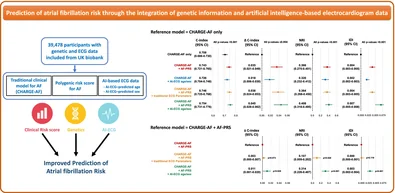

## 1 Introduction

Atrial fibrillation (AF) is the most common sustained arrhythmia and is associated with an increased risk of stroke, heart failure, cognitive decline, and mortality [1, 2]. Accurate prediction of AF is therefore essential for effective prevention and timely intervention. Clinical risk scores, such as the CHARGE‐AF score, were developed to estimate future AF risk using routinely available clinical factors including age, anthropometric measures, blood pressure, and cardiovascular history [3]. While practical and easy to implement, their predictive accuracy remains limited, particularly among younger individuals or those without overt cardiovascular comorbidities [4, 5].

To address these limitations, genetic approaches have been introduced. AF‐specific polygenic risk score (AF‐PRS), derived from genome‐wide association studies (GWAS), captures inherited susceptibility to AF and provides incremental improvements in risk prediction beyond clinical factors [6, 7]. However, AF‐PRS alone offers only modest gains in discrimination and does not fully reflect subclinical pathophysiologic changes [4].

Artificial intelligence–enabled electrocardiogram (AI‐ECG) analysis has recently emerged as a promising digital biomarker [8]. By inferring biological age and sex from raw ECG signals, AI‐ECG can detect subtle electrical remodeling not appreciable to clinicians [9, 10]. These features have been associated with AF incidence and broader cardiovascular outcomes, suggesting their potential role in risk stratification [11].

Despite these advances, most prior studies have evaluated clinical risk scores, AF‐PRS, and AI‐ECG features in isolation [3, 4, 10]. In practice, risk assessment is likely to be most effective when these complementary domains—clinical risk factors, genetic predisposition, and subclinical electrical signatures—are integrated [12]. Therefore, the present study aimed to develop and validate an enhanced AF prediction model by combining AI‐ECG–derived age and sex features with AF‐PRS and established clinical risk factors, building upon the foundation of the CHARGE‐AF score.

## 2 Methods

### 2.1 Study Population

This study was conducted using data from the UK Biobank, a large‐scale prospective cohort of approximately 500 000 individuals aged 40 to 69 years recruited from 22 assessment centers across the United Kingdom between 2006 and 2010. Participants underwent extensive baseline phenotyping, including questionnaires, physical measurements, and biological samples [13]. In addition, digital 12‐lead resting electrocardiograms (ECGs) were acquired for a subset of participants during the imaging visit, which commenced in 2014 [14].

**FIGURE 1:**
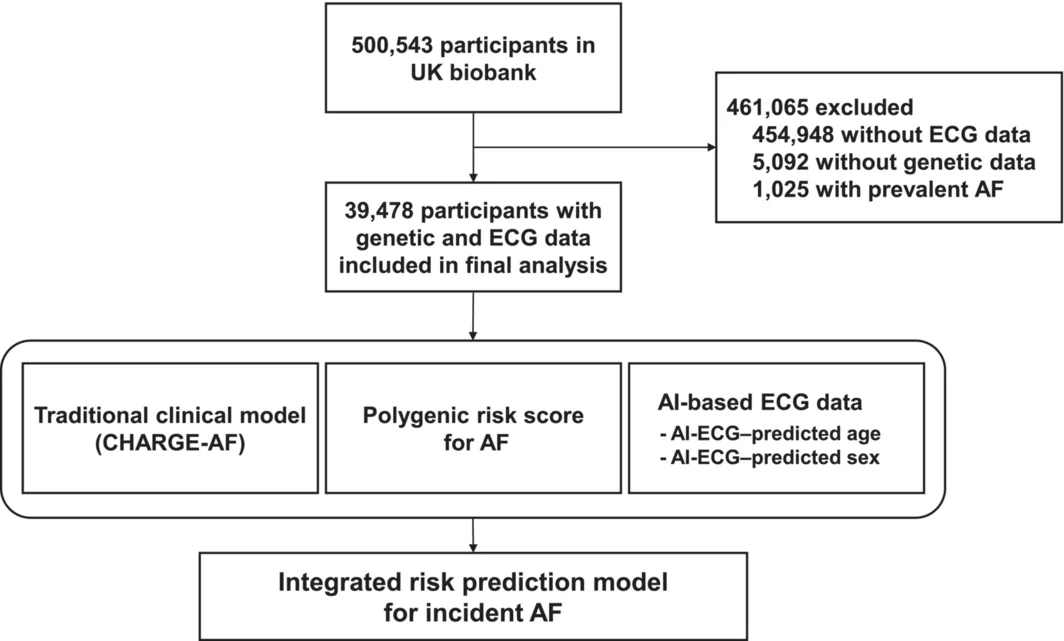
Study population and flowchart. AF, atrial fibrillation; AI, artificial intelligence; ECG, electrocardiogram; PRS, polygenic risk score.

For the present analysis, inclusion criteria were: (1) availability of baseline ECG recordings, (2) genetic data that passed quality control, and (3) complete information on clinical variables required to calculate the CHARGE‐AF score. Participants with a history of AF at baseline were excluded. AF at baseline was defined using linked hospital episode statistics, self‐reported medical history, or evidence of AF on the baseline ECG. After these exclusions, 39 478 participants without prior AF were included in the analytic cohort. The study population flowchart is presented in Figure 1.

### 2.2 Ascertainment of Atrial Fibrillation

Incident AF events were identified by linkage to national hospital admission data, death registries, and repeat ECGs [13]. The diagnosis of AF was based on International Classification of Diseases (ICD‐10) codes (I48.x) recorded in hospital episode statistics, or as an underlying or contributing cause of death in the death registry [15]. Participants were followed from the date of baseline assessment until the earliest of the following: (1) first diagnosis of AF, (2) death, (3) loss to follow‐up, or (4) the last available update of registry data (maximum 6.7 years).

### 2.3 Clinical Risk Assessment

The CHARGE‐AF score was selected as the clinical reference model for AF prediction [3, 16, 17]. This score incorporates widely available variables, including age, height, weight, systolic and diastolic blood pressure, current smoking status, antihypertensive medication use, history of myocardial infarction, and history of heart failure. All covariates were derived from standardized baseline assessments within the UK Biobank, including self‐administered questionnaires, physical examinations, and linked health records.

### 2.4 Genetic Risk Assessment

Genotyping in the UK Biobank was performed using the UK BiLEVE or UK Biobank Axiom arrays, followed by imputation to a reference panel combining the UK10K and 1000 Genomes Phase 3 datasets [18]. Standard quality control procedures were applied centrally, including filtering for missingness, Hardy–Weinberg equilibrium, and minor allele frequency thresholds.

For this study, AF‐PRS was constructed based on single nucleotide polymorphisms (SNPs) previously identified in large‐scale GWAS of AF [6, 19, 20]. The score was calculated as a weighted sum of risk alleles, with each allele weighted by its effect size (log‐odds ratio) reported in the discovery GWAS. The AF‐PRS was standardized to a mean of zero and a standard deviation of one within the study cohort, allowing direct comparison of effect sizes per one standard deviation increase in genetic risk.

Of note, this AF‐PRS was developed independently of the UK Biobank, using a meta‐analysis of three external GWAS comprising 48 646 AF cases and 256 971 controls [20]. There was no sample overlap between the external discovery cohorts and the UK Biobank, thereby minimizing potential overfitting and ensuring external validity. Furthermore, it has been validated in large‐scale, population‐based cohorts including the 100 000 Genomes Project (100KGP), and demonstrated robust predictive accuracy across different ancestries and recruitment frameworks.

### 2.5 AI‐ECG Feature Extraction

Baseline 12‐lead ECGs were digitally recorded in the UK Biobank using a standardized 10‐s acquisition protocol. Raw ECG waveforms were analyzed with previously developed residual network (ResNet)–based convolutional neural network (CNN) models [9, 21, 22, 23, 24].

The AI‐ECG–predicted age was obtained using the ResNet‐based model originally developed by Lima et al. [22]. In our previous work, we externally validated this model across four independent multinational cohorts: CODE‐15% (*n* = 345 779), PhysioNet (*n* = 21 799), Sami‐Trop (*n* = 1 631), and UK Biobank (*n* = 45 595) [22, 23]. The AI‐ECG age gap, treated as a continuous variable, was defined as the AI‐ECG–predicted age minus the chronological age. For binary analyses, a positive AI‐ECG age gap was defined as AI‐ECG–predicted age > chronological age; a negative gap, as AI‐ECG–predicted age ≤ chronological age.

For AI‐ECG sex prediction, we developed a ResNet‐based model trained on the Medical Information Mart for Intensive Care (MIMIC‐IV) cohort (*n* = 13 628) in our prior study [9, 24]. In the same study, this model was externally validated in two large multinational cohorts, CODE‐15% (*n* = 345 779) and UK Biobank (*n* = 45 595) [24]. This model generated the probability that the AI‐ECG–predicted sex differed from the reported biological sex. The output value ranged from 0 to 1, representing the likelihood of sex mismatch (i.e., the probability that a male would be classified as female or vice versa). A probability > 0.5 was defined as AI‐ECG–predicted sex mismatch, whereas ≤ 0.5 indicated sex match.

Of note, no model training was performed using UK Biobank ECGs; the UK Biobank cohort was utilized solely for external validation in prior publications [23, 24] and for application in the present analysis. By employing independently developed and externally validated models, this study ensured that the AI‐derived features incorporated into the risk prediction framework were based on reproducible and generalizable tools.

### 2.6 Statistical Analysis

Baseline characteristics of the study population were summarized using standard descriptive statistics. Continuous variables were expressed as means with standard deviations or as medians with interquartile ranges, depending on their distribution, while categorical variables were presented as counts with corresponding percentages. Comparisons between groups were made using χ^2^ tests for categorical variables and either Student's *t*‐tests or Wilcoxon rank‐sum tests for continuous variables, as appropriate.

For the analysis of incident AF, time‐to‐event methods were applied using Cox proportional hazards regression. Hazard ratios (HRs) with 95% confidence intervals (CIs) were calculated for each of the primary risk factors of interest, namely the AF‐PRS, the AI‐ECG age gap, and the AI‐ECG sex mismatch probability. All models were adjusted for age, sex, and established clinical covariates.

To evaluate incremental predictive performance, three sequential risk models were constructed. The first model consisted of the CHARGE‐AF score alone, representing a purely clinical model. The second model incorporated both clinical factors and the polygenic risk score. The third and final model further added the AI‐ECG features, specifically the age gap (treated as a continuous variable and defined as the AI‐ECG–predicted age minus the chronological age) and the sex mismatch (modeled as a binary variable, derived from the probability that the AI‐ECG–predicted sex differed from the reported biological sex; values > 0.5 were classified as sex mismatch and ≤ 0.5 as sex match), to the clinical and genetic predictors.

Model performance was assessed across several domains. Discrimination was evaluated using Harrell's concordance index (C‐index) [25]. Reclassification was examined through the continuous net reclassification improvement (NRI) and the integrated discrimination improvement (IDI) [26]. Finally, the practical benefit of each model was examined using decision curve analysis, which estimates net benefit across a range of clinically relevant decision thresholds [27].

All analyses were performed with the R statistical software package (version 4.5.1, R Foundation for Statistical Computing, Vienna, Austria). A two‐sided *p*‐value of less than 0.05 was considered to indicate statistical significance.

## 3 Results

### 3.1 Baseline Characteristics

Over a median follow‐up of 2.7 years (interquartile range [IQR], 1.7–4.2; maximum, 6.7 years), 533 participants (1.3%) developed incident AF. Baseline characteristics of the study population according to AF occurrence are presented in Table 1. Participants who subsequently developed AF were significantly older (mean age 68.2 vs. 63.9 years, *p* < 0.001) and more frequently male (67.7% vs. 47.7%, *p* < 0.001). They also had higher systolic blood pressure, greater height, greater body weight, and increased body mass index (all *p* < 0.001). Comorbid conditions were more prevalent in the AF group, including hypertension (43.7% vs. 25.6%), diabetes mellitus (6.9% vs. 2.9%), heart failure (4.5% vs. 2.4%), prior ischemic stroke (2.1% vs. 0.5%), myocardial infarction (5.6% vs. 1.8%), malignancy (20.6% vs. 14.6%), and chronic kidney disease (3.0% vs. 1.4%) (all *p* < 0.01). In terms of risk profiles, participants with incident AF had higher CHA_2_DS_2_‐VASc scores, CHARGE‐AF scores, and AF‐PRS (all *p* < 0.001). Electrocardiographic parameters also differed significantly: The AF group showed longer PQ intervals (*p* = 0.006), prolonged QRS durations (*p* < 0.001), and greater QTc intervals (*p* < 0.001). Importantly, AI‐ECG–derived features demonstrated marked differences, with individuals who developed AF exhibiting higher AI‐ECG–predicted age (*p* < 0.001), a more frequent positive AI‐ECG age gap (*p* < 0.001), larger age gap values (*p* = 0.013), and increased sex mismatch probability (*p* = 0.007) as well as higher rates of predicted sex mismatch (*p* = 0.007) compared with those without AF.

**TABLE 1:** Baseline characteristics of the study population according to incidence of AF.

	Overall (*n* = 39 478)	No incident AF (*n* = 38 945)	Incident AF (*n* = 533)	*p*
Age, years	64.0 ± 7.7	63.9 ± 7.7	68.2 ± 6.2	< 0.001
Male	18 939 (48.0%)	18 578 (47.7%)	361 (67.7%)	< 0.001
Systolic BP, mmHg	138.3 ± 18.6	138.3 ± 18.6	142.7 ± 19.6	< 0.001
Diastolic BP, mmHg	79.1 ± 10.1	79.1 ± 10.1	79.7 ± 11.0	0.196
Height, cm	169.1 ± 9.2	169.0 ± 9.2	172.0 ± 8.8	< 0.001
Weight, kg	76.0 ± 15.2	75.9 ± 15.2	82.0 ± 16.3	< 0.001
Body mass index, kg/m^2^	26.5 ± 4.4	26.5 ± 4.4	27.6 ± 4.7	< 0.001
Former smoker	13 395 (33.9%)	13 159 (33.8%)	236 (44.3%)	< 0.001
Current smoker	1 420 (3.6%)	1 397 (3.6%)	23 (4.3%)	0.3484
**Comorbidity**				
Hypertension	10 212 (25.9%)	9 979 (25.6%)	233 (43.7%)	< 0.001
Diabetes	1 185 (3.0%)	1 148 (2.9%)	37 (6.9%)	< 0.001
Heart failure	962 (2.4%)	938 (2.4%)	24 (4.5%)	0.004
History of ischemic stroke	209 (0.5%)	198 (0.5%)	11 (2.1%)	< 0.001
History of MI	718 (1.8%)	688 (1.8%)	30 (5.6%)	< 0.001
History of malignancy	5 793 (14.7%)	5 683 (14.6%)	110 (20.6%)	< 0.001
COPD	82 (0.2%)	79 (0.2%)	3 (0.6%)	0.100
CKD or ESRD	574 (1.5%)	558 (1.4%)	16 (3.0%)	0.009
**Clinical risk score/Polygenic risk score**				
CHA_2_DS_2_‐VASc score	1.0 [1.0, 2.0]	1.0 [1.0, 2.0]	2.0 [1.0, 3.0]	< 0.001
CHARGE‐AF score	12.4 [11.7, 13.1]	12.4 [11.7, 13.1]	13.1 [12.6, 13.6]	< 0.001
AF polygenic risk score	0.07 ± 0.91	0.07 ± 0.91	0.52 ± 0.95	< 0.001
**ECG‐derived parameters**				
ECG *p* duration, ms	97.9 ± 16.3	97.9 ± 16.3	99.2 ± 20.4	0.172
ECG PQ interval, ms	164.9 ± 27.3	164.9 ± 27.2	169.1 ± 32.2	0.006
ECG QRS duration, ms	88.5 ± 14.1	88.5 ± 14.0	92.0 ± 16.0	< 0.001
ECG QTc interval, ms	421.1 ± 25.6	421.0 ± 25.5	426.4 ± 33.1	< 0.001
AI‐ECG–predicted age, years	63.4 ± 8.4	63.4 ± 8.4	68.4 ± 7.3	< 0.001
AI‐ECG age gap, years^a^	−0.58 ± 8.03	−0.60 ± 8.03	0.22 ± 7.51	0.013
AI‐ECG sex mismatch probability^b^	0.15 ± 0.27	0.15 ± 0.27	0.19 ± 0.29	0.007

*Note:* Values are presented as mean ± standard deviation (SD), number (percentage), or median [interquartile range], as appropriate. Group comparisons were conducted using Fisher's exact tests for categorical variables and Student's *t*‐tests or Wilcoxon rank‐sum tests for continuous variables.

Abbreviations: AF, atrial fibrillation; AI, artificial intelligence; BP, blood pressure; CKD, chronic kidney disease; COPD, chronic obstructive pulmonary disease; ECG, electrocardiogram; ESRD, end‐stage renal disease; MI, myocardial infarction.

^a^ AI‐ECG age gap was defined as AI‐ECG–predicted age minus chronological age.

^b^ AI‐ECG sex mismatch probability was defined as the probability that the AI‐ECG–predicted sex differed from the reported biological sex, ranging from 0 to 1.

### 3.2 Distribution of Clinical, Genetic, and AI‐ECG Predictors of AF

**FIGURE 2:**
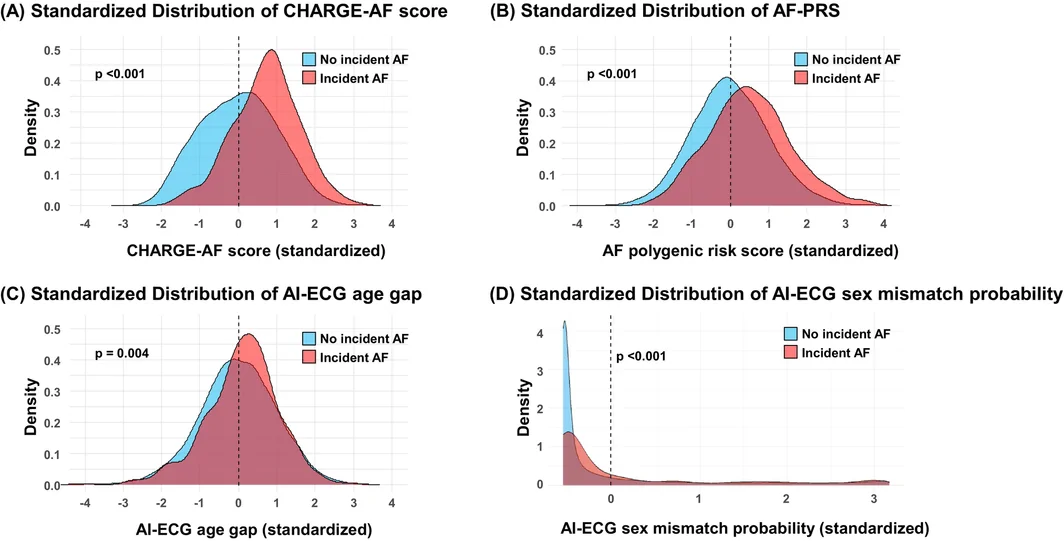
Standardized distributions of AF risk predictors among participants with and without incident AF. Panels show the distributions of (A) CHARGE‐AF score, (B) AF‐PRS, (C) AI‐ECG age gap, and (D) AI‐ECG sex mismatch probability during follow‐up. All variables were standardized (z‐scores) within the analytic sample. Vertical dashed lines indicate the standardized mean (0) for each predictor. AF, atrial fibrillation; AI, artificial intelligence; ECG, electrocardiogram; PRS, polygenic risk score.

Standardized density distributions of key AF risk predictors are shown in Figure 2, providing a visualization of how these variables differ across the entire spectrum of values, rather than only at the mean. This approach allows detection of distributional shifts and divergence patterns that simple group averages may obscure. Compared with participants without AF, those who developed incident AF showed rightward‐shifted distributions of both the CHARGE‐AF score and the AF‐PRS, reflecting higher cumulative clinical and genetic risk burdens (both *p* < 0.001). For AI‐ECG features, AF cases exhibited a higher frequency of positive age gaps (Panel C, *p* = 0.004) and substantially elevated sex mismatch probabilities (Panel D, *p* < 0.001). Notably, the distribution of AI‐ECG sex mismatch probability demonstrated more pronounced separation in the upper tail, capturing distinctions that may not be evident from mean comparisons alone.

### 3.3 Cumulative Incidence of AF According to AI‐ECG Features

**FIGURE 3:**
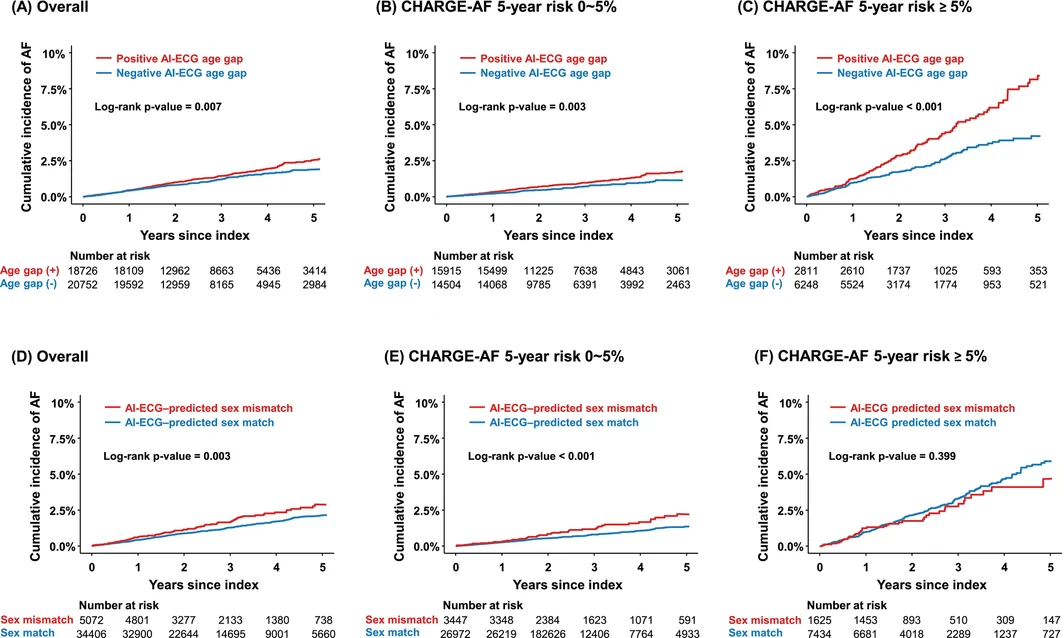
Cumulative incidence of atrial fibrillation (AF) stratified by AI‐ECG features and CHARGE‐AF 5‐year risk. Kaplan–Meier curves show the cumulative incidence of AF according to the AI‐ECG age gap (Panels A–C) and AI‐ECG–predicted sex mismatch (Panels D–F). (A, D) Overall cohort; (B, E) participants with a CHARGE‐AF 5‐year risk < 5%; (C, F) participants with a CHARGE‐AF 5‐year risk ≥ 5%. A positive AI‐ECG age gap was defined as AI‐ECG–predicted age greater than chronological age, and a negative age gap as AI‐ECG–predicted age less than or equal to chronological age. AI‐ECG–predicted sex match was defined as a mismatch probability ≤ 0.5, and sex mismatch as a probability > 0.5. AF, atrial fibrillation; AI, artificial intelligence; ECG, electrocardiogram.

Kaplan–Meier analyses demonstrated that AI‐ECG–derived features were significantly associated with incident AF (Figure 3). Participants with a positive age gap (defined as AI‐ECG–predicted age greater than chronological age) had a higher cumulative incidence of AF compared with those with a negative age gap (AI‐ECG–predicted age less than or equal to chronological age; log‐rank *p* = 0.007; Figure 3A). This pattern persisted when stratifying by clinical risk categories based on the CHARGE‐AF 5‐year risk score: Among individuals classified as lower‐risk (CHARGE‐AF 5‐year risk < 5%), a positive age gap was associated with greater AF incidence (*p* = 0.003; Figure 3B), and the association was even more pronounced in the higher‐risk group (CHARGE‐AF 5‐year risk ≥ 5%; *p* < 0.001; Figure 3C).

Similarly, AI‐ECG–derived sex prediction provided additional discrimination. Participants with predicted sex mismatch (AI‐ECG–predicted sex different from biological sex, defined as a mismatch probability greater than 0.5) experienced higher AF incidence than those with predicted sex match (AI‐ECG–predicted sex same as biological sex, defined as a mismatch probability of 0.5 or less; log‐rank *p* = 0.003; Figure 3D). This relationship was consistent in the lower‐risk subgroup (CHARGE‐AF 5‐year risk < 5%; *p* < 0.001; Figure 3E). However, in the higher‐risk subgroup (CHARGE‐AF 5‐year risk ≥ 5%), cumulative incidence curves for predicted sex match versus mismatch did not differ significantly (*p* = 0.399; Figure 3F).

### 3.4 Independent Contributions of Genetic and AI‐ECG Predictors Beyond CHARGE‐AF

Building upon the Kaplan–Meier analyses, multivariable Cox regression models were used to evaluate the independent contribution of AF predictors (Table 2).

**TABLE 2:** Hazard ratios for incident AF according to sequentially augmented risk prediction models.

	Covariates	Variable	HR (95% CI)^a^	*p*
**Model 1**	**CHARGE‐AF only**	CHARGE‐AF	2.44 (2.22–2.68)	< 0.001
**Model 2**	**CHARGE‐AF + AF‐PRS**	CHARGE‐AF	2.45 (2.24–2.69)	< 0.001
		AF‐PRS	1.62 (1.49–1.76)	< 0.001
**Model 3**	**CHARGE‐AF + AF‐PRS + AI‐ECG age/sex (continuous)**	CHARGE‐AF	2.65 (2.40–2.92)	< 0.001
		AF‐PRS	1.61 (1.48–1.76)	< 0.001
		AI‐ECG age gap	1.37 (1.24–1.51)	< 0.001
		AI‐ECG sex mismatch probability	1.04 (0.96–1.13)	0.322
**Model 4**	**CHARGE‐AF + AF‐PRS + AI‐ECG age/sex (categorical)**	CHARGE‐AF	2.44 (2.22–2.68)	< 0.001
		AF‐PRS	1.61 (1.49–1.76)	< 0.001
		Positive AI‐ECG age gap	1.79 (1.51–2.14)	< 0.001
		AI‐ECG–predicted sex mismatch	1.15 (0.92–1.44)	0.229

Abbreviations: AF, atrial fibrillation; AI, artificial intelligence; ECG, electrocardiogram; PRS, polygenic risk score.

^a^ Hazard ratios (HR) are presented per 1‐standard deviation (SD) increase for continuous variables (CHARGE‐AF, AF‐PRS, AI‐ECG age gap, and AI‐ECG sex mismatch probability), and as HR for binary variables (positive AI‐ECG age gap, AI‐ECG–predicted sex mismatch; reference = negative AI‐ECG age gap or AI‐ECG–predicted sex match). All models were adjusted for the covariates listed within each model specification.

In the base model including only the CHARGE‐AF score, each 1‐SD increase was associated with more than a twofold higher AF risk (HR 2.44, 95% CI: 2.22–2.68; *p* < 0.001). Adding the AF‐PRS (Model 2) added complementary information to CHARGE‐AF in multivariable models, with AF‐PRS independently associated with incident AF (HR 1.62, 95% CI: 1.49–1.76; *p* < 0.001), while the effect of CHARGE‐AF remained robust (HR 2.45, 95% CI: 2.24–2.69; *p* < 0.001).

When AI‐ECG features were incorporated as continuous variables (Model 3), both CHARGE‐AF (HR 2.65, 95% CI: 2.40–2.92; *p* < 0.001) and AF‐PRS (HR 1.61, 95% CI: 1.48–1.76; *p* < 0.001) remained strong predictors, and the AI‐ECG age gap independently predicted AF (HR 1.37, 95% CI: 1.24–1.51; *p* < 0.001). In contrast, the AI‐ECG sex mismatch probability showed no significant association (HR 1.04, 95% CI: 0.96–1.13; *p* = 0.322). In categorical analyses (Model 4), a positive AI‐ECG age gap (reference = negative age gap) was strongly associated with AF risk (HR 1.79, 95% CI: 1.51–2.14; *p* < 0.001). However, AI‐ECG–predicted sex mismatch (reference = predicted sex match) did not reach statistical significance (HR 1.15, 95% CI: 0.92–1.44; *p* = 0.229).

**FIGURE 4:**
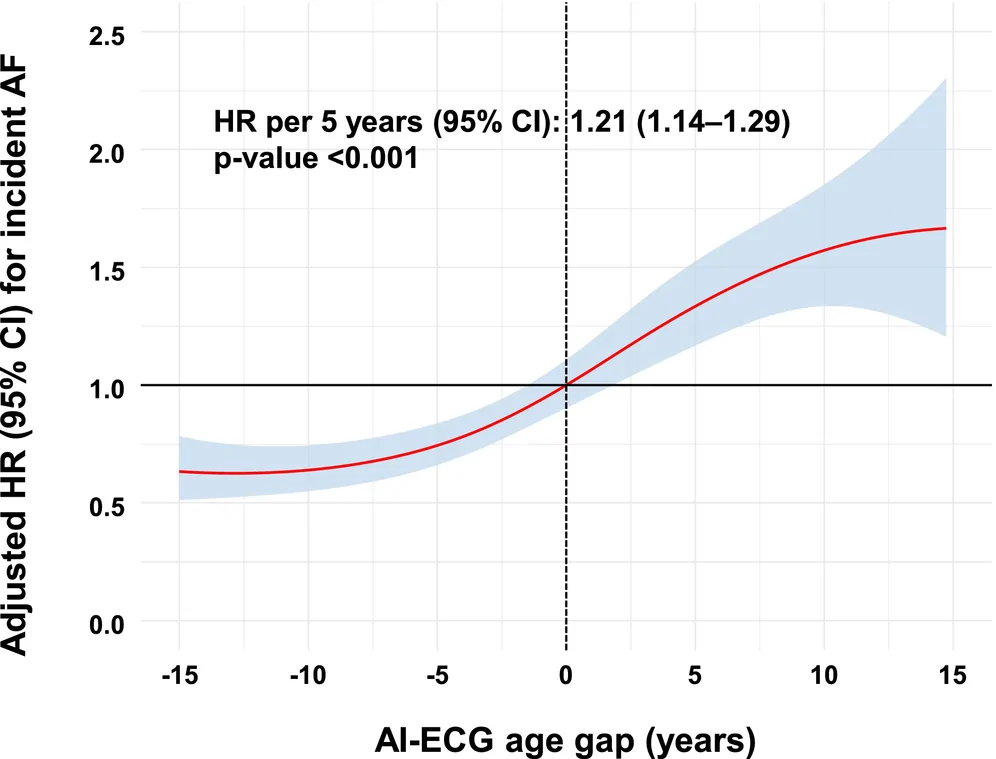
Spline curve showing the association between AI‐ECG age gap and the risk of incident AF. The hazard ratios (HRs) with 95% confidence intervals (CIs) were derived from a Cox proportional hazards model, adjusted for CHARGE‐AF score and AF‐PRS. AF, atrial fibrillation; AI, artificial intelligence; ECG, electrocardiogram; PRS, polygenic risk score.

The prognostic relevance of the AI‐ECG age gap was further assessed using restricted cubic spline analysis (Figure 4). The risk of AF increased steadily as the AI‐ECG age gap widened, demonstrating a graded and approximately linear relationship. Each 5‐year increase in the AI‐ECG age gap was associated with a 21% higher risk of AF (HR 1.21, 95% CI: 1.14–1.29; *p* < 0.001), even after adjustment for CHARGE‐AF and AF‐PRS. These findings indicate the incremental and independent prognostic value of the AI‐ECG age gap, complementing both clinical (CHARGE‐AF) and genetic (AF‐PRS) risk stratification.

### 3.5 Incremental Predictive Value of Genetic and AI‐ECG Features

**FIGURE 5:**
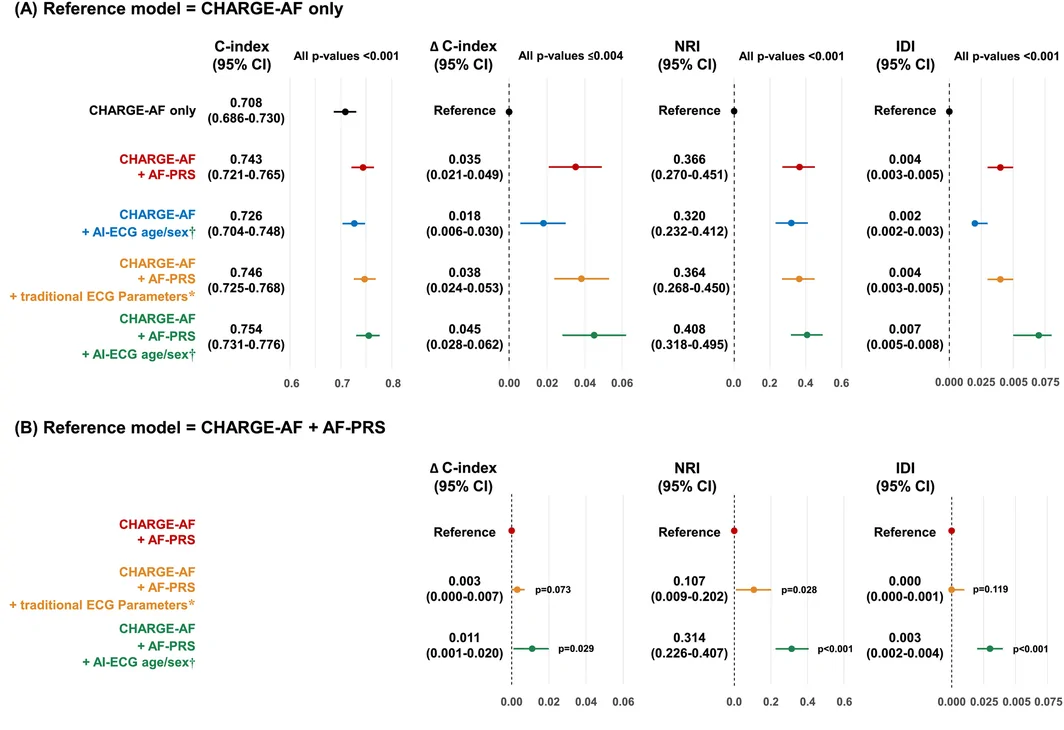
Discrimination indices for incident AF according to sequentially augmented risk prediction models. (A) CHARGE‐AF model as the reference. (B) CHARGE‐AF + AF‐PRS model as the reference. *Traditional ECG parameters included P wave duration, PQ interval, QRS duration, and QTc interval. †AI‐ECG features included the AI‐ECG age gap (analyzed as a continuous variable) and the AI‐ECG sex mismatch (applied as a binary variable using a probability threshold of 0.5). AF, atrial fibrillation; AI, artificial intelligence; ECG, electrocardiogram; PRS, polygenic risk score; NRI, net reclassification improvement; IDI, integrated discrimination improvement.

Sequential augmentation of risk prediction models demonstrated stepwise improvements in the discrimination of incident AF. When the CHARGE‐AF model was used as the reference (Figure 5A), this baseline model yielded a C‐index of 0.708 (95% CI: 0.686–0.730). The addition of AF‐PRS significantly improved discrimination (C‐index 0.743, 95% CI: 0.721–0.765; ΔC‐index 0.035, 95% CI: 0.021–0.049; *p* < 0.001), with corresponding improvements in reclassification metrics (NRI 0.366, 95% CI: 0.270–0.451; *p* < 0.001; IDI 0.004, 95% CI: 0.003–0.005; *p* < 0.001). Further inclusion of traditional ECG parameters (P wave duration, PQ interval, QRS duration, and QTc interval) yielded only minimal additional gain (C‐index 0.746, 95% CI: 0.725–0.768). By contrast, incorporation of AI‐ECG features, namely the age gap (as a continuous variable) and the sex mismatch (as a binary variable defined by a mismatch probability threshold of 0.5), led to more substantial improvements (C‐index 0.754, 95% CI: 0.731–0.776; ΔC‐index 0.045, 95% CI: 0.028–0.062; NRI 0.408, 95% CI: 0.318–0.495; IDI 0.007, 95% CI: 0.005–0.008; all *p* < 0.001).

When the CHARGE‐AF + AF‐PRS model was used as the reference (Figure 5B), adding traditional ECG parameters provided only statistically nonsignificant or marginal improvement (ΔC‐index 0.003, 95% CI: 0.000–0.007; *p* = 0.073; NRI 0.107, 95% CI: 0.009–0.202; *p* = 0.028; IDI 0.000, 95% CI: 0.000–0.001; *p* = 0.119). In contrast, further incorporation of AI‐ECG features achieved a meaningful enhancement in risk prediction (ΔC‐index 0.011, 95% CI: 0.001–0.020; *p* = 0.029), with consistent improvements in both NRI (0.314, 95% CI: 0.226–0.407; *p* < 0.001) and IDI (0.003, 95% CI: 0.002–0.004; *p* < 0.001).

When the CHARGE‐AF + AI‐ECG model was used as the reference (Figure S1), the addition of AF‐PRS significantly improved discrimination (ΔC‐index 0.027, 95% CI: 0.015–0.040; *p* < 0.001), reclassification (NRI 0.350, 95% CI: 0.253–0.434; *p* < 0.001), and integrated discrimination (IDI 0.004, 95% CI: 0.003–0.006; *p* < 0.001).

Taken together, these results demonstrate that genetic information (AF‐PRS) and AI‐ECG features provide complementary and additional predictive value beyond established clinical risk factors, whereas traditional ECG parameters offer only limited improvement.

### 3.6 Decision Curve Analysis for Practical Benefit

**FIGURE 6:**
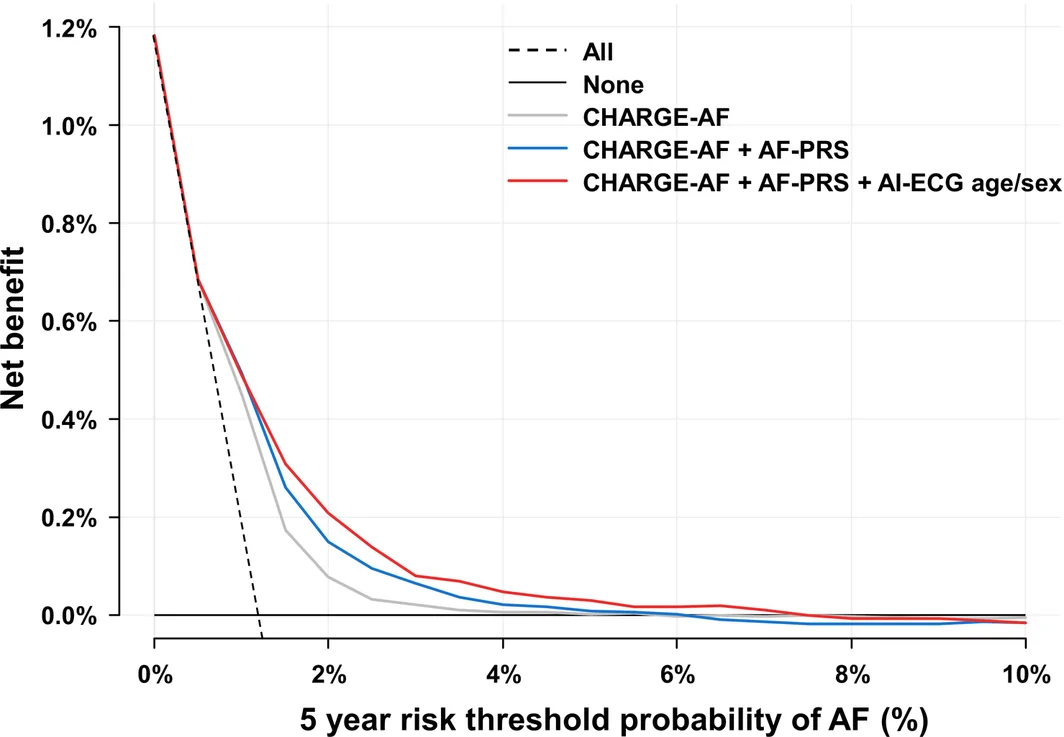
Decision curve analysis for incident AF according to sequentially augmented risk prediction models. AF, atrial fibrillation; AI, artificial intelligence; ECG, electrocardiogram; PRS, polygenic risk score.

Decision curve analysis was conducted to evaluate the potential clinical usefulness of sequentially augmented AF prediction models (Figure 6). The baseline CHARGE‐AF model alone demonstrated only marginal net benefit across most clinically relevant 5‐year risk thresholds. When AF‐PRS was added to the CHARGE‐AF model, the net benefit curve shifted upward, indicating a meaningful gain in decision‐making utility. Importantly, further incorporation of AI‐ECG features (AI‐ECG age gap and AI‐ECG sex mismatch) produced the greatest overall improvement. This integrative model yielded clear net benefit, particularly within the lower predicted risk range of approximately 1%–7%, a threshold range where preventive interventions would be most clinically impactful. Collectively, these findings suggest that incorporating genetic and AI‐ECG features into conventional clinical risk models enhances the practical applicability of AF risk prediction.

## 4 Discussion

### 4.1 Main Findings

In this study, we showed that sequential augmentation of risk prediction models with genetic and AI‐ECG features significantly improved the discrimination of incident AF. The CHARGE‐AF model, which incorporates conventional clinical risk factors, served as the baseline comparator. The addition of an AF‐PRS led to meaningful improvements in model discrimination and reclassification. Importantly, incorporation of AI‐ECG features, including the ECG age gap and AI‐ECG–predicted sex mismatch, provided further incremental predictive value, exceeding the gains observed with traditional ECG parameters. Decision curve analysis further demonstrated that the integrated models yielded greater net clinical benefit across a range of risk thresholds, particularly at lower predicted risks, highlighting their potential clinical utility.

### 4.2 Complementary Role of Genetic Risk Information

Genetic predisposition has been consistently shown in prior studies to improve AF risk prediction [4, 7, 28], and our findings reinforce the substantial contribution of AF‐PRS when added to the CHARGE‐AF model. Importantly, the strength of AF‐PRS lies not only in its independent predictive capacity but also in its complementary contribution within a multimodal risk prediction framework. In an analysis using the CHARGE‐AF + AI‐ECG model as the reference, AF‐PRS significantly improved discrimination and reclassification, demonstrating incremental predictive value even after AI‐ECG features were incorporated. Together, these findings support a synergistic framework in which genetic and AI‐derived information complement conventional clinical risk scores to refine AF risk stratification.

### 4.3 Clinical Significance of AI‐ECG Features

The ECG age gap, representing the discrepancy between biological age inferred from the ECG and chronological age, may reflect subclinical cardiac remodeling or electrophysiological aging [9, 23, 29, 30]. Similarly, AI‐ECG–predicted sex mismatch indicates deviation from expected sex‐specific ECG patterns. Such mismatch may represent a marker of subtle structural or electrophysiological remodeling that shifts the ECG phenotype away from typical sex‐specific patterns, potentially through alterations in atrial size, conduction properties, and repolarization vectors. Subtle deviations in ECG phenotypes, while not captured by conventional ECG parameters, may be identified by AI models and could signal latent structural or electrical alterations associated with AF susceptibility [24, 31]. It should be emphasized, however, that the biological mechanisms linking these AI‐derived ECG features to AF risk remain incompletely understood, and our interpretation is necessarily speculative in the absence of direct experimental validation.

Taken together, these AI‐derived ECG markers appear to convey prognostic information for AF beyond what is accessible from conventional ECG parameters. From a mechanistic perspective, these AI‐derived signals likely represent early manifestations of atrial remodeling and biological aging that precede the clinical onset of AF [29, 30]. Clinically, their integration into risk models consistently improved discrimination and reclassification, supporting their potential as practical tools for more precise risk stratification. Importantly, AI‐ECG features offered substantial additional predictive value not only beyond established clinical risk scores but also beyond genetic predisposition. This suggests that AI‐ECG captures a dimension of risk that is complementary yet distinct from both conventional clinical determinants and inherited susceptibility. As such, AI‐ECG features may serve as integrative biomarkers that link mechanistic insights with actionable risk prediction, thereby providing a unique opportunity to enhance early identification, targeted surveillance, and ultimately prevention of AF.

### 4.4 Comparison With Conventional ECG Parameters

Traditional ECG intervals, including P wave duration, PQ interval, QRS duration, and QTc interval, have long been regarded as markers of atrial and ventricular conduction properties [32, 33]. However, in our study, their incremental contribution to AF risk prediction was negligible when added to established clinical and genetic models. This observation suggests that while conventional ECG parameters capture aspects of overt electrical conduction, they may not fully encompass the subtle and diffuse electrophysiological alterations that precede clinical AF. In contrast, AI‐derived ECG features provided substantial incremental value in risk prediction beyond both clinical risk scores and genetic predisposition. Unlike fixed interval measurements, AI‐ECG models integrate high‐dimensional patterns across the entire waveform, thereby detecting latent signatures of cardiac remodeling or biological aging that remain inaccessible to conventional parameters [10, 34, 35]. These findings emphasize the limitation of relying solely on standard ECG intervals for risk stratification and indicate the potential of AI‐enabled approaches to capture additional prognostic signals from the same routinely acquired test.

### 4.5 Clinical Applicability and Future Directions

The ultimate value of any risk prediction model lies in its translation into clinical practice. Our findings suggest that integration of genetic and AI‐ECG features with established risk scores meaningfully enhances risk stratification for AF, with potential implications for individualized prevention and management strategies. Decision curve analysis indicates that such integrative models demonstrate greater net clinical benefit across a broad range of risk thresholds, particularly at lower predicted risks where early intervention may have the greatest impact.

The potential clinical value of refined prediction in this relatively low absolute‐risk range lies primarily in better targeting of AF screening and preventive strategies. Individuals identified as being at higher risk could be considered for more intensive rhythm surveillance, such as repeated ECG assessment or intermittent/wearable ECG monitoring, to facilitate earlier detection of asymptomatic or paroxysmal AF. Enhanced risk stratification may also help prioritize more focused assessment and management of modifiable AF‐promoting factors, including hypertension, obesity, sleep apnea, physical inactivity, and excessive alcohol consumption. Thus, rather than directly altering treatment recommendations, the model may refine current approaches to AF screening and risk‐factor modification by identifying individuals in whom these strategies could be more selectively applied. Prospective studies are needed to establish appropriate risk thresholds and determine whether such risk‐guided strategies improve clinical outcomes.

The predictive contributions of genetic and AI‐ECG markers may operate over different temporal scales. AF‐PRS reflects relatively stable lifelong genetic susceptibility, whereas AI‐ECG features may capture the current electrophysiological state of the heart, including ongoing electrical remodeling, biological aging, and subclinical structural changes. Thus, AI‐ECG may be particularly informative for short‐ to intermediate‐term AF risk and may complement the more stable risk information provided by genetic predisposition. Whether this incremental predictive value persists over longer time horizons, such as 10 years, requires further validation with extended follow‐up.

Beyond clinical, genetic, and AI‐ECG information, cardiac structural parameters, particularly measures of left atrial size and function, may provide complementary information for AF risk prediction by directly reflecting atrial structural remodeling. Incorporating these measures into risk prediction models could further improve risk stratification and help determine whether AF‐PRS and AI‐ECG features provide incremental prognostic information beyond structural cardiac remodeling assessed by imaging. Future studies incorporating structural parameters are therefore warranted.

Prior AI‐ECG studies have primarily focused on directly predicting AF from raw waveforms in an end‐to‐end manner. While effective, such models often provide limited mechanistic interpretability and may face challenges in generalizability across diverse cohorts. In contrast, our approach leveraged intermediate AI‐derived features (ECG age gap and sex mismatch probability) that not only improved risk prediction but also offered mechanistic insights into the pathways linking ECG‐derived signatures to AF development. Furthermore, because age and sex are universal and biologically grounded characteristics, the use of such interpretable features enhances the scalability and adaptability of the models across populations and healthcare settings. This feature‐based strategy may therefore provide greater transparency, clinical acceptance, and extensibility compared with direct AF prediction models.

From a practical perspective, AI‐ECG algorithms can be embedded into routine ECG platforms to enable automated and scalable risk assessment without additional testing. Integrating AI‐derived markers with AF‐PRS and clinical factors may advance precision cardiology by tailoring prevention and surveillance to individual profiles. Future studies, including prospective trials, are needed to determine how such models can be incorporated into screening strategies and whether their use improves clinical outcomes.

## 5 Limitations

Several limitations warrant consideration. First, this study was conducted only within a single large cohort (UK Biobank), which represents an important limitation. While the findings provide valuable insights, the external validity and generalizability remain inherently constrained. Independent validation in other large‐scale, population‐based cohorts that incorporate clinical, genetic, and ECG data will be essential to confirm the robustness and applicability of our results across diverse populations and healthcare settings. Second, the relatively short median follow‐up of 2.7 years limits direct assessment of long‐term AF risk, and the present findings should not be directly extrapolated to 10‐year risk without further validation. Third, detailed cardiac structural parameters, including measures of left atrial remodeling and left ventricular hypertrophy, were not uniformly available in the analytic cohort. Therefore, we could not determine the extent to which AF‐PRS and AI‐ECG features provide incremental predictive information beyond imaging‐derived measures of cardiac structural remodeling. Fourth, while the AI‐ECG features showed strong predictive value, the underlying biological mechanisms remain incompletely understood and merit further mechanistic investigation. Fifth, decision curve analysis provides only an estimate of practical benefit, and real‐world implementation studies are necessary to determine their actual impact on patient outcomes. Finally, although genetic and AI‐ECG data can enhance prediction, issues of accessibility, cost, and integration into routine care may limit broad applicability, particularly in resource‐limited settings. These challenges should be carefully addressed in future translational efforts.

## 6 Conclusion

In this large, population‐based cohort study, we demonstrated that integrating AI‐ECG features with polygenic and clinical risk factors enhances prediction of incident AF. Among the AI‐ECG parameters, the AI‐ECG age gap was independently associated with future AF risk, while the AI‐ECG sex mismatch probability did not reach statistical significance as an individual predictor. Nevertheless, the incorporation of both AI‐ECG features alongside genetic and clinical risk factors improved overall model performance, yielding superior discrimination and improved risk reclassification compared with models based on clinical factors alone or combined clinical and genetic information. Decision curve analysis further confirmed that the integrated model provided greater net clinical benefit across a broad spectrum of decision thresholds.

These findings indicate the value of combining digital biomarkers derived from routine ECGs with genetic information to refine AF risk prediction beyond traditional clinical scores. By capturing inherited susceptibility together with subclinical electrophysiologic alterations, this integrated approach may support earlier identification of high‐risk individuals, facilitate more personalized preventive strategies, and advance precision cardiology in AF.

## Author Contributions

P.‐S.Y. interpreted data, performed statistical analyses, generated figures and tables, and wrote the manuscript. H.P., O.‐S.K., J.‐W.P., D.H.K., H.T.Y., T.‐H.K., J.‐S.U., and B.J. contributed to the interpretation and manuscript revisions. H.‐N.P. designed and coordinated the study, interpreted data, and contributed to the writing of the manuscript. All authors have read and approved the manuscript for publication.

## Funding

This work was supported by the National Research Foundation of Korea (RS‐2025‐16064327).

## Conflicts of Interest

The authors declare no conflicts of interest.

## Supporting information


**Figure S1:** Discrimination indices for incident AF with the CHARGE‐AF + AI‐ECG age/sex model as the reference. AI‐ECG features included the AI‐ECG age gap (analyzed as a continuous variable) and the AI‐ECG sex mismatch (applied as a binary variable using a probability threshold of 0.5). AF, atrial fibrillation; AI, artificial intelligence; ECG, electrocardiogram; PRS, polygenic risk score; NRI, net reclassification improvement; IDI, integrated discrimination improvement.

## Data Availability

The data that support the findings of this study are available from the UK Biobank. Access to these data is granted to bona fide researchers through an approved application process (www.ukbiobank.ac.uk). The analytic methods and AI‐ECG model code used for feature derivation are available from the corresponding author upon reasonable request.
