# Supplementary material for: Prediction of Atrial Fibrillation Risk Through the Integration of Genetic Information and Artificial Intelligence‐Based Electrocardiogram Data
Source: J Arrhythm. 2026 Sep 24;42(5):e70463. doi: 10.1002/joa3.70463 (PMC13602817; doi:10.1002/joa3.70463)
Supplement: Supplementary file 1 [file JOA3-42-e70463-s001.docx]

**Supplemental material**

**Supplemental Figure 1.** Discrimination indices for incident AF with the CHARGE-AF + AI-ECG age/sex model as the reference. AI-ECG features included the AI-ECG age gap (analyzed as a continuous variable) and the AI-ECG sex mismatch (applied as a binary variable using a probability threshold of 0.5). AF, atrial fibrillation; AI, artificial intelligence; ECG, electrocardiogram; PRS, polygenic risk score; NRI, net reclassification improvement; IDI, integrated discrimination improvement.
